# Structural and Electrochemical Evolution of Nickel Sulfides During Alkaline Hydrogen Evolution Reaction

**DOI:** 10.1002/cssc.202501880

**Published:** 2025-12-19

**Authors:** Sina Haghverdi Khamene, Noëlle van Dalen, Mariadriana Creatore, Mihalis N. Tsampas

**Affiliations:** ^1^ Department of Applied Physics and Science Education Eindhoven University of Technology Eindhoven the Netherlands; ^2^ DIFFER – Dutch Institute for Fundamental Energy Research Eindhoven the Netherlands; ^3^ Eindhoven Institute for Renewable Energy Systems (EIRES) Eindhoven the Netherlands

**Keywords:** electrochemical activation, hydrogen evolution reaction (HER), nickel sulfides, phase transformation, sulfur leaching

## Abstract

Nickel Sulfides have emerged as promising electrocatalysts for alkaline hydrogen evolution reaction (HER) due to their cost‐effectiveness and high catalytic activity. While growing research has focused on the initial catalyst design, less attention has been paid to structural and electrochemical modifications during prolonged HER operation. Understanding these transformations is essential for developing more active and stable nickel sulphide‐based HER catalysts. This study investigated the post‐HER evolution of various nickel sulphide crystalline catalysts, including NiS, NiS_2_, Ni_3_S_2_, and Ni_3_S_4_, after prolonged cyclic voltammetry (CV) cycling and constant current polarization. Upon 500 CV sweeps, Raman spectroscopy confirmed structural phase transformation of all nickel Sulfides toward Ni_3_S_2_, i.e., the most HER‐active phase, irrespective of their initial chemical composition. This electrochemical activation process led to an improvement in electrochemical surface area and charge–transfer properties. Moreover, the kinetic analysis indicated a shift in the rate‐determining step from a Volmer‐limited mechanism to a mixed Volmer‐Heyrovsky pathway, contributing to enhanced HER kinetics. Sulfur leaching was identified as a key factor in this transformation, facilitating surface restructuring and exposure of active Ni sites to the electrolyte. Importantly, post‐stability characterization confirmed that leaching occurs predominantly during initial activation and ceases thereafter, with no further structural changes over prolonged operation.

## Graphical abstract

Post‐HER surface restructuring of nickel Sulfides (Ni_x_S_y_) through Sulfur leaching results in the formation of catalytically active Ni_3_S_2_, the most HER‐active nickel sulphide phase. The electrochemical activation process enhances hydrogen evolution performance while exhibiting self‐limiting behavior, ensuring long‐term catalyst stability in alkaline media.

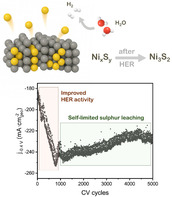

## 1 Introduction

The global transition to renewable energy sources is essential for reducing carbon emissions and mitigating climate change [1, 2]. Among the sustainable energy carriers, green hydrogen, produced through water electrolysis powered by renewable energy, has emerged as a promising alternative [3, 4]. However, the overall efficiency of green hydrogen relies on the performance of water electrolysis, where the hydrogen evolution reaction (HER) is a key step [5, 6]. In alkaline water electrolysis, HER involves a multistep pathway, including the initial water dissociation step (Volmer step), which is often the rate‐limiting step due to the high energy barrier required to break the strong H—O bond in water [7]. The reaction is then followed by either the Volmer–Heyrovsky or Volmer–Tafel pathway. In the Heyrovsky step, the adsorbed hydrogen intermediate reacts with an electron and a water molecule to form molecular hydrogen. In contrast, in the Tafel step, two adsorbed hydrogen atoms directly recombine to generate H_2_ [8, 9]. Since the Volmer step involves water dissociation, its high energy barrier often limits reaction efficiency, particularly in alkaline conditions where proton availability is lower compared to acidic HER [7, 10]. Accordingly, shifting the rate‐limiting step to either the Heyrovsky or Tafel step leads to a lower overall energy requirement and enhanced HER performance.

The efficiency of HER is largely determined by the catalyst's ability to facilitate both water dissociation and hydrogen desorption [11, 12]. Among the various non‐precious metal electrocatalysts, nickel Sulfides (Ni_*x*_S_*y*_) have gained significant attention due to their cost‐effectiveness, high HER activity, and tunable physical and chemical properties, making them promising candidates for alkaline HER applications [13, 14, 15]. In these compounds, nickel atoms are known as the primary active sites, facilitating the adsorption of hydrogen intermediates during HER, which is essential for the Volmer step because of Ni's ability to bind hydrogen effectively [16, 17, 18]. Sulfur, on the other hand, is known to play a key role in modifying the electron distribution in Ni_*x*_S_*y*_. This electronic tuning weakens overly strong Ni–H binding, facilitating balanced hydrogen adsorption and desorption [19, 20, 21, 22]. Such synergy between Ni and S not only enhances the Volmer step, where water dissociation occurs, but also improves subsequent hydrogen evolution steps, creating complementary active centers for the overall HER pathway. In line with this, Ding et al. [17] demonstrated that interfacial Ni sites in nickel Sulfides are primarily responsible for water dissociation and OH adsorption, while interfacial S sites facilitate hydrogen adsorption and subsequent H_2_ evolution, highlighting the cooperative role of both elements in enhancing HER activity.

The Ni_*x*_S_*y*_ phases range from stoichiometric compounds such as NiS, NiS_2_, Ni_3_S_2_, and Ni_3_S_4_ to amorphous forms, each displaying distinct electronic structures, conductivity, and catalytic activity [23, 24, 25]. To further improve the HER performance of nickel Sulfides, various strategies have been employed, including phase engineering [26, 27, 28], heteroatom doping [29, 30, 31], heterostructure construction [32, 33, 34], vacancy engineering [35, 36, 37, 38, 39, 40, 41], etc. Most of these studies focus on modulating the electronic properties and optimizing the active site distribution to achieve improved charge transfer kinetics, enhanced hydrogen adsorption/desorption equilibrium, and increased electrochemically active surface area (ECSA). Despite these advancements, critical gaps remain in understanding the evolution of the structural and electrochemical properties of nickel Sulfides upon HER conditions. Nickel Sulfides are known to undergo structural transformations and surface modifications under prolonged HER operation, which can significantly influence their catalytic activity and stability [17, 42, 43]. While many studies have focused on initial catalyst design and engineering strategies, fewer efforts have been dedicated to systematically investigating the long‐term behavior of nickel Sulfides after the hydrogen evolution reaction. Understanding how phase stability and charge‐transfer characteristics change during HER is essential for developing efficient electrocatalysts. These transformations can either enhance catalytic activity by exposing new active sites or lead to degradation through structural instability [44]. Addressing these knowledge gaps will enable strategies for improving the long‐term performance of nickel sulphide‐based HER catalysts.

To address this, four of the most stable nickel sulphide crystalline phases, including NiS, NiS_2_, Ni_3_S_2_, and Ni_3_S_4_, were synthesized via the Sulfurization of 3D micropillar‐based Ni electrodes (Ni Veco), a substrate geometry we previously investigated for alkaline water electrolysis applications [45, 46]. The selection of these phases was not intended to determine which phase exhibits superior HER activity but rather to gain a more comprehensive understanding of the structural and electrochemical modifications that nickel Sulfides undergo during prolonged HER operation. By systematically comparing these phases, this study aims to provide a broader perspective on phase evolution, electrochemical restructuring, and HER kinetics in nickel Sulfides post‐HER. To achieve this, all synthesized nickel Sulfides were subjected to prolonged cyclic voltammetry (CV) cycling as an electrochemical activation step. Furthermore, the impact of electrochemical activation on the HER mechanism was investigated by analyzing the evolution of kinetic parameters, providing insights into potential shifts in the rate‐determining step and catalytic pathways.

## 2 Experimental

### 2.1 Sulfurization of Nickel Electrodes

The Sulfurization of Ni electrodes was performed following protocols developed in parallel with this work [46]. Briefly, the process was conducted in a Mellen SV series tube furnace. Ni Veco substrates (Veco Precision BV, ZP200617B, 0.6 mm thickness, 99.99 wt.% purity) [45, 47] were utilized for both Sulfurization and subsequent physicochemical and electrochemical analyses. The electrodes feature uniformly shaped and dimensioned micropillars and holes, facilitating gas bubble release and improving mass transport during electrochemical operation. The electrodes were initially cleaned by sonication for 20 min each in deionized water, acetone, and isopropyl alcohol sequentially. The cleaned nickel substrate was then centered within a quartz tube inside the furnace, which was heated to the desired temperature at a rate of 20°C min^−1^. After reaching the set temperature, the system was held steady for at least 30 min to achieve a uniform temperature distribution. An H_2_S/Ar gas stream (Linde Gas Benelux BV, 10% H_2_S) was then introduced at a flow rate of 100 sccm to form NiS_2_, Ni_3_S_2_, and NiS phases at temperatures of 250°C, 300°C, and 500°C, respectively. For the Ni_3_S_4_ phase, the temperature was maintained at 300°C with a reduced flow rate of 40 sccm. Each Sulfurization step lasted 1 h, yielding film thicknesses of 1.8 ± 0.9 µm (Ni_3_S_2_), 1.7 ± 1.1 µm (NiS_2_), 15.0 ± 9.0 µm (NiS), and 1.7 ± 0.2 µm (Ni_3_S_4_). Following the Sulfurization, the gas flow was stopped, and the system was cooled down at an approximate rate of 5°C min^−1^ using a fan. The samples were then immediately moved to a glovebox to maintain them under controlled conditions.

### 2.2 Physicochemical Characterization

Raman spectroscopy was performed using a Renishaw inVia system with a 514 nm excitation laser at 1% intensity under ambient conditions. Spectra were recorded over a 100–800 cm^−1^ Raman shift range, with each spectrum acquired over 20 s and accumulated 10 times. Grazing‐incidence X‐ray diffraction (GIXRD) patterns were acquired using a Bruker D8 diffractometer equipped with a Cu K*α* radiation source (*λ* = 1.54060 Å). Data were collected with a step size of 0.03° and an integration time of 14 s per step. X‐ray photoelectron spectroscopy (XPS) was conducted with a Thermo Scientific KA1066 spectrometer using a monochromatic Al K*α* X‐ray source. Binding energies were calibrated by referencing the S 2p_3/2_ peak of the S^2−^ species to 161.7 eV to account for valence electron effects. Finally, scanning electron microscopy (SEM) [ZEISS Sigma, 3 kV acceleration voltage] was employed to investigate the microstructure and morphology of the synthesized electrodes, before and after HER.

### 2.3 Electrochemical Characterization

Electrochemical measurements were carried out in a single‐compartment, three‐electrode cell (C‐A‐STD_EC‐150, Redox.me) equipped with modified Teflon walls for enhanced chemical resistance, using a CompactStat (Ivium Technologies) potentiostat. Samples were mounted in a holder with a 1 cm^2^ aperture (Redox.me) and used as the working electrode. A reversible hydrogen electrode (RHE) [Mini‐HydroFlex, Gaskatel] served as the reference electrode, and a Ni mesh (Veco Precision BV, ZP10034, 0.3 mm thick, 99.99 wt.%) was used as the counter electrode. The electrolyte was a 1.0 M KOH (Sigma–Aldrich) commercial solution.

The evaluation protocol initiated with cyclic voltammetry (CV) conducted in the non‐Faradaic range (0.1–0.2 V vs. RHE) at multiple scan rates of 10, 20, 30, 40, and 50 mV·s^−1^, each repeated three times, to determine the ECSA of pristine electrodes. Electrochemical impedance spectroscopy (EIS) was performed at −0.4 V versus RHE over 0.1–10,000 Hz, with an AC amplitude of 10 mV. The resulting Nyquist plots were analyzed to extract charge transfer resistance (*R*_CT_) values. HER activity was then evaluated via linear sweep voltammetry (LSV) from −0.4 to 0.0 V versus RHE at 1 mV·s^−1^, along with Tafel slope determination. Tafel slopes were determined using the approach suggested by O. van der Heijden et al. [48], where local slopes were calculated over small overpotential intervals (5 mV) and plotted as a function of overpotential. Electrodes were then electrochemically activated through 500 CV sweeps in the −0.4–0.0 V versus RHE range at 50 mV·s^−1^. After activation, HER activity and Tafel slopes were re‐evaluated using LSV under the same conditions. An automatic iR‐compensation, accounting for 80% of the ohmic resistance determined from EIS, was applied during all CV and LSV measurements. ECSA and EIS measurements were also repeated to assess changes in active surface area and charge‐transfer properties resulting from the activation process. The ECSA was determined using the double‐layer capacitance method. The capacitive current was obtained by averaging the anodic and cathodic currents at the midpoint of the non‐Faradaic range. These values were then plotted against the scan rate, and the double‐layer capacitance was extracted as the slope of the fitted linear trend. Finally, the ECSA was calculated by normalizing the double‐layer capacitance with the specific capacitance of nickel sulphide. Given the variability of specific capacitance values in literature, an average value of 40 μF·cm^−2^ was employed for nickel sulphide in alkaline solution [49]. Finally, chronopotentiometric measurements were conducted to assess the long‐term stability of the catalysts. Following electrochemical activation through 500 CV sweeps, the stability was assessed over 100 h at constant current densities of 10 and 100 mA·cm^−2^.

## 3 Results and Discussion

### 3.1 Characterization of Pristine Electrodes

**FIGURE 1:**
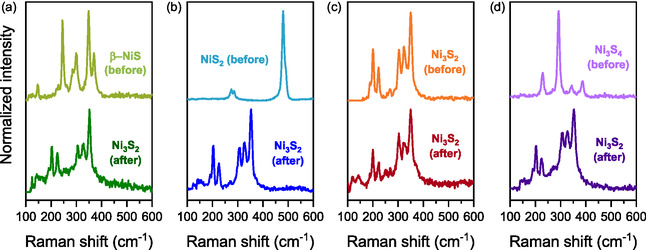
Raman spectra of (a) NiS, (b) NiS_2_, (c) Ni_3_S_2_, and (d) Ni_3_S_4_ before and after electrochemical activation.

Raman spectroscopy was employed to verify the phase composition of the synthesized nickel sulphide samples, building upon our previous investigations. The Raman spectra, presented in Figure 1, distinctly confirm the formation of the four nickel sulphide phases, each exhibiting characteristic vibrational modes associated with their respective crystalline structures. Figure 1a displays a Raman spectrum with vibrational modes at 146, 221, 244, 284, 298, 349, and 371 cm^−1^, characteristic of the β‐NiS phase. These modes correspond to the lattice vibrations and phonon interactions within the Ni‐S framework, validating the formation of β‐NiS [23, 50]. In Figure 1b, the observed Raman bands at 275, 285, and 480 cm^−1^ are attributed to the Tg, Eg, and Ag vibration modes of NiS_2_, respectively [51, 52]. Similarly, Figure 1c exhibits Raman peaks at 188, 201, 224, 304, 325, and 350 cm^−1^, corresponding to the two A1 and four E Raman modes of Ni_3_S_2_ [53]. Finally, Figure 1d depicts Raman peaks at 224, 287, 337, and 380 cm^−1^, which are assigned to the Eg, Tg, Tg, and Ag phonon modes of Ni_3_S_4_, further confirming its formation [23, 54]. The GIXRD patterns (Figure S1) confirm the formation of the intended nickel sulphide phases as well, in agreement with Raman spectroscopy results. However, interpretation of the diffraction data is challenged by overlapping signals from the underlying Ni substrate, which are especially prominent in some samples, and by the inherently low intensity and higher background noise associated with the 3D architecture of the substrates. Due to these limitations, Raman spectroscopy was employed as the primary tool for phase identification and comparison, while GIXRD serves as a complementary technique to support phase assignment [46].

**FIGURE 2:**
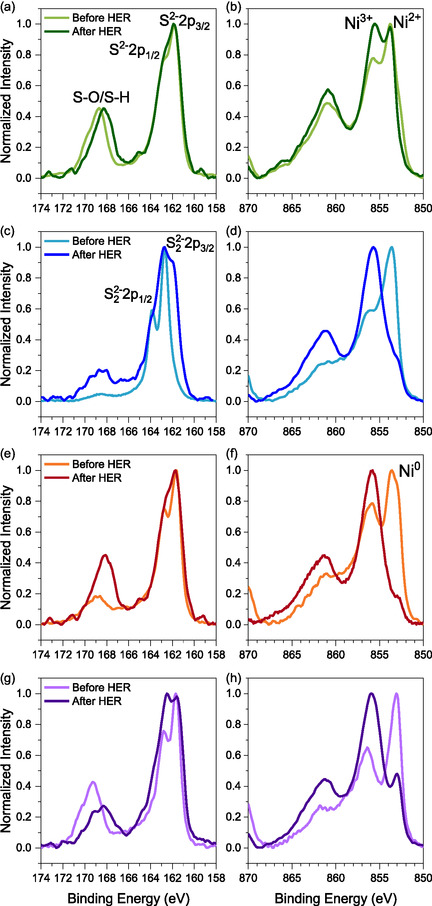
High‐resolution XPS spectra of (a,b) NiS, (c,d) NiS_2_, (e,f) Ni_3_S_2_, and (g,h) Ni_3_S_4_ before and after electrochemical activation. The spectra correspond to S 2p (a,c,e,g) and Ni 2p (b,d,f,h) region.

X‐ray photoelectron spectroscopy (XPS) was used to examine the surface chemistry of the pristine nickel sulphide electrodes. Survey spectra (Figures S2 and S5) confirm the presence of Ni, S, and adventitious C, with minor oxygen signals attributed to surface oxidation during air exposure. To gain insights into the bonding structures, oxidation states, and possible transformations upon HER, high‐resolution XPS spectra of the S 2p, Ni 2p, and O 1s regions were analyzed for all four samples (Figures 2 and S6). The S 2p spectra display well‐resolved S 2p_3/2_ and S 2p_1/2_ doublets, with NiS (Figure 2a), Ni_3_S_2_ (Figure 2e), and Ni_3_S_4_ (Figure 2g) exhibiting peaks at ∼161.7 and ∼162.8 eV characteristic of S^2−^ species [55, 56, 57], whereas NiS_2_ (Figure 2c) shows a positive shift (∼162.0 and ∼163.8 eV) indicative of S_2_^2−^ species. The Ni 2p spectra exhibit the characteristic Ni 2p_3/2_ and Ni 2p_1/2_ spin–orbit doublets with associated satellites. NiS (Figure 2b) and NiS_2_ (Figure 2d) show features consistent with Ni^2+^ in Ni‐S bonding, with slight binding energy shifts in NiS_2_ due to the S_2_^2−^ environment. Ni_3_S_2_ (Figure 2f) presents a broader, slightly lower‐energy main peak (∼852.5–853.0 eV), reflecting partial Ni–Ni metallic character as Ni^0^ signal, while Ni_3_S_4_ (Figure 2h) displays mixed‐valence features consistent with Ni^2+^/Ni^3+^ [58, 59, 60, 61]. This provides the reference spectral signatures for subsequent comparison with post‐HER data.

### 3.2 Electrochemical Activation and Impact on Catalytic Properties

#### 3.2.1 HER Performance Evolution

**FIGURE 3:**
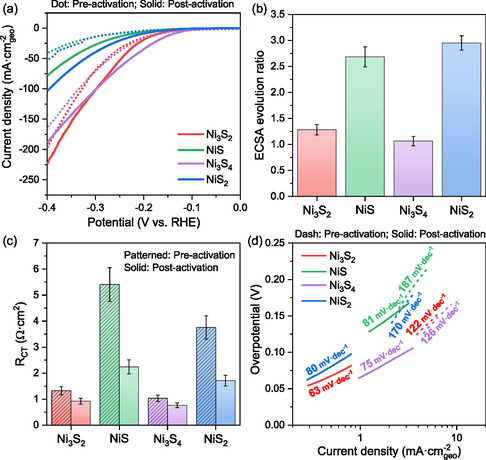
(a) LSV plots, (b) ECSA evolution ratio, (c) Charge‐transfer resistance values derived from EIS Nyquist plots, and (d) Tafel slopes of different nickel Sulfides before and after electrochemical activation (500 CV sweeps) in 1.0 M KOH.

As detailed in the Experimental Section, the nickel sulphide electrodes underwent electrochemical activation through 500 CV cycles [62]. The impact of this step on HER activity is illustrated in the LSV plots before and after activation (Figure 3a). A notable enhancement in HER performance was observed across all phases following electrochemical activation. The current densities recorded at −0.4 V vs. RHE increased from −41, −52, −165, and −197 mA·cm^−2^ (before activation) to −80, −104, −188, and −225 mA·cm^−2^ (after activation) for NiS, NiS_2_, Ni_3_S_4_, and Ni_3_S_2_, respectively. This corresponds to an activation‐induced improvement of approximately 100% for NiS and NiS_2_, while Ni_3_S_4_ and Ni_3_S_2_ exhibited a more moderate enhancement of ∼14%. These findings demonstrate that the magnitude of improvement upon activation is phase‐dependent. The substantial enhancement in NiS and NiS_2_ suggests significant electrochemical restructuring and active site generation, whereas the smaller improvement for Ni_3_S_2_ and Ni_3_S_4_ indicates limited surface transformation.

#### 3.2.2 Structural and Surface Evolution Post‐HER

The Raman spectra recorded after electrochemical activation (Figure 1) show a notable phase transformation across all nickel sulphide samples, with all phases converging to Ni_3_S_2_ upon activation. This structural evolution is characterized by the emergence of distinct Ni_3_S_2_ vibrational modes at approximately 188, 201, 224, 304, 325, and 350 cm^−1^, confirming the dominance of this phase [53]. A similar CV‐induced phase transformation from NiS to Ni_3_S_2_ was previously reported by X. Ding et al*.* [17], but here, this transformation is observed across all nickel sulphide phases, indicating a more generalized electrochemically driven restructuring process. This electrochemically induced phase evolution explains the enhanced HER activity observed after activation. However, notably, the Ni_3_S_2_ sample itself underwent a moderate activation, suggesting that phase transformation is not the sole contributing factor to performance improvement.

**FIGURE 4:**
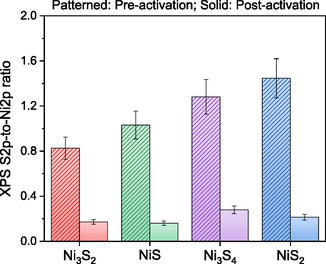
XPS‐derived S2p‐to‐Ni2p ratio of different nickel Sulfides before and after electrochemical activation.

To further elucidate the structural and chemical transformations induced by electrochemical activation, XPS was conducted to examine changes in the elemental composition, oxidation states, and surface chemistry of the nickel sulphide electrodes after HER. Figure 4 displays the Ni/S ratio derived from the Ni 2p and S 2p spectra, providing insights into Sulfur retention and depletion across different phases. As shown, the initial S/Ni ratios varied among the pristine nickel sulphide phases (ranging from 0.82 to 1.45), reflecting inherent compositional differences.

However, upon electrochemical activation, a notable reduction in Sulfur content was observed across all samples, with the final S/Ni ratio ranging between 0.17 and 0.27. This Sulfur depletion, commonly referred to as Sulfur leaching, is a well‐documented phenomenon in nickel Sulfides under electrochemical conditions and is often associated with the formation of nickel‐rich sulphide surfaces or surface oxidation [63, 64, 65]. Interestingly, even Ni_3_S_2_, which retains its overall phase composition upon activation, undergoes a significant decrease in Sulfur content in the near‐surface region. This suggests that surface restructuring and partial deSulfurization occur independently of overall phase stability, further affecting the electrochemical properties of the electrodes.

To gain a detailed understanding of the surface chemistry modifications induced by electrochemical activation, high‐resolution XPS spectra of the post‐activation samples were compared to those recorded before HER (Figure 2). The S 2p spectra of NiS, Ni_3_S_2_, and Ni_3_S_4_ (Figure 2a,e,g) exhibit a slight modification, characterized by an increase in the relative intensity of the S^2−^ 2p_1/2_ peak after activation. In general, the S 2p_3/2_ peak is attributed to metal‐Sulfur bonds, whereas the S 2p_1/2_ component, located at higher binding energy, is often associated with Sulfur species of lower coordination, which are linked to the presence of Sulfur vacancies [66, 67]. The observed shift in the intensity ratio between the S 2p_1/2_ and S 2p_3/2_ peaks indicates that selective Sulfur loss occurs within the catalyst structure during HER operation, consistent with earlier reports [68].

For Ni_3_S_4_ (Figure 2g), this increase is more pronounced, accompanied by the appearance of a new feature at higher binding energies, suggesting the partial reorganization into new S_2_^2−^ states upon HER. However, this transformation was not detectable in Raman spectra, indicating that this transformation is confined to the near‐surface region detectable by XPS, rather than being persistent throughout the entire material. For NiS_2_ (Figure 2c), a distinct transformation from S_2_^2−^ to S^2−^ is evident, occurring alongside the phase transition to Ni_3_S_2_ upon activation. If oxidation were responsible, a similar change would be expected pre‐HER; however, the S‐O signal appears at 168–170 eV, well separated from the S^2−^/S_2_^2−^ envelope at ∼161.5–164 eV. The coexistence of both Sulfur species in the XPS spectra indicates that this transformation remains incomplete, with S^2−^ and S_2_^2−^ still present at the surface. This finding disagrees with Raman spectroscopy results, which confirm a full bulk transformation to Ni_3_S_2_. This discrepancy can be explained by the different probing depths of the two techniques. Raman spectroscopy captures the bulk structure, confirming full transformation to Ni_3_S_2_, whereas XPS is surface‐sensitive (a few nanometers) and reveals residual S_2_^2−^ species confined to the near‐surface region. These residual species may persist due to incomplete surface conversion or dynamic exchange with Sulfur‐containing species in the electrolyte during HER, which would not be detected by Raman [69, 70]. This highlights the influence of electrochemical cycling on surface Sulfur chemistry, where near‐surface species may persist even after deeper structural stabilization, contributing to the observed spectral differences.

In addition, scanning electron microscopy (SEM) was conducted on all samples before and after HER to examine potential morphological modifications upon electrochemical activation (Figure S7 and S8). The SEM images exhibited no remarkable difference in morphology between the pristine and post‐HER states, indicating that the activation‐induced transformations occurred primarily at the atomic level rather than through macroscale structural reorganization. In all cases, the Ni_*x*_S_*y*_ crystallites remained visible with different sizes depending on the synthesis conditions, with no indication of morphological reconstruction.

#### 3.2.3 Evolution of ECSA and Charge‐Transfer Properties

The effect of electrochemical activation on phase transformation and surface chemistry has been discussed, demonstrating its role in enhancing the intrinsic catalytic activity of nickel sulphide electrodes. However, beyond intrinsic activity, the evolution of electrochemically active site density plays a key role in improving HER performance. To quantify these effects, the ECSA was evaluated before and after activation (Figure 3c, S9, and S10), with a higher ECSA ratio indicating a more significant structural modification. The ECSA values were derived from the double‐layer capacitance (*C*_dl_) obtained by linear fitting of the capacitive current (Δ*j*/2 at 0.15 V vs. RHE) against scan rate, with representative plots and fitting uncertainties provided in the Supporting Information. Among the samples, NiS and NiS_2_ exhibit the most substantial increases in ECSA, with ∼3.0 ± 0.3‐fold improvement. This enhancement correlates with their complete phase transformation to Ni_3_S_2_ upon activation, as confirmed by Raman and XPS analysis, and is further driven by Sulfur leaching, which exposes additional Ni active sites to the electrolyte [24, 63]. Compared with NiS and NiS_2_, the ECSA changes upon activation were less pronounced for Ni_3_S_2_ and Ni_3_S_4_, showing only ∼1.2 ± 0.1‐fold increases, which suggests limited surface restructuring and higher structural stability. In addition to the phase stability of Ni_3_S_2_, this suggests that Ni_3_S_4_ possesses a robust structure with a more stable spinel‐like crystal structure, reducing the extent of active site exposure upon activation [71, 72]. This correlation strengthens the conclusion that electrochemical restructuring and phase transformation drive the exposure of new active sites.

The effect of electrochemical activation on charge‐transfer kinetics was further analyzed by evaluating the electrochemical impedance spectroscopy (EIS) results (Figure S11). The charge‐transfer resistance (*R*_CT_), which reflects the efficiency of electron transfer at the electrode–electrolyte interface, showed a notable decrease upon activation, following the same trend as HER activity and ECSA evolution (Figure 3d). The most substantial improvements were observed in NiS and NiS_2_, which exhibited 2.4‐fold and 2.2‐fold reductions in *R*_CT_, respectively, correlating with their high HER activity and large ECSA increases. The decrease in *R*_CT_ can be attributed to enhanced conductivity and improved electron transport pathways, primarily driven by phase transformation to Ni_3_S_2_ [65, 73]. Besides, Sulfur leaching plays a crucial role by exposing catalytically active Ni sites, while simultaneously removing insulating Sulfur species, thereby optimizing electron flow pathways [74, 75, 76]. Even in Ni_3_S_2_, which is already the stable bulk phase, a further 1.5‐fold decrease in *R*_CT_ through localized Sulfur removal at the surface suggests that surface restructuring during activation optimizes charge‐transfer characteristics. This effect can be linked to localized Sulfur removal at the surface, which generates Sulfur vacancies that act as electronic conductivity enhancers. These vacancies create more conductive Ni‐rich domains and reduce localized charge trapping, thereby enhancing charge carrier mobility [77, 78]. In addition, Ni_3_S_4_, while exhibiting the lowest improvement (1.4‐fold), still demonstrates a notable reduction in charge transfer resistance, suggesting that its surface undergoes meaningful modifications upon activation. This aligns with its moderate but significant increase in ECSA, indicating that although Ni_3_S_4_ maintains a relatively stable structure, electrochemical activation still enhances its charge‐transfer kinetics, albeit to a lesser extent than the other phases. Together, Figure 3a–c provide a coherent picture linking phase transformation, surface restructuring, and electronic conductivity to improvement in HER performance.

#### 3.2.4 HER Kinetics and Mechanistic Insights

**FIGURE 5:**
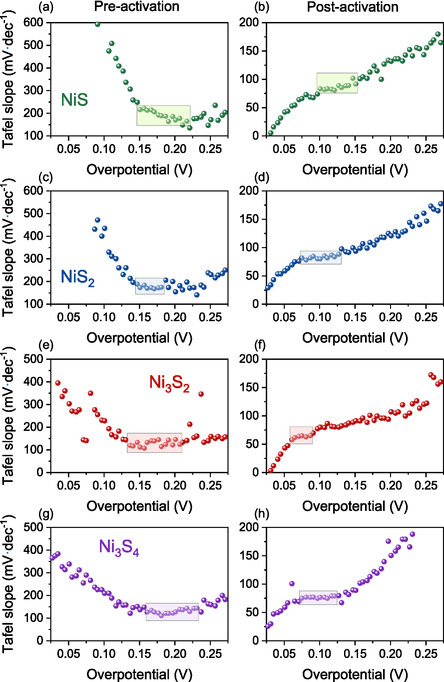
Tafel slope values calculated over 5 mV overpotential intervals and plotted as a function of the average overpotential for (a,b) NiS, (c,d) NiS_2_, (e,f) Ni_3_S_2_, and (g,h) Ni_3_S_4_ samples. Panels (a,c,e,g) represent data before electrochemical activation, while panels (b,d,f,h) correspond to after activation.

The electrochemical activation‐induced transformations in phase composition, surface chemistry, ECSA, and *R*_CT_ have been systematically examined, all of which contribute to the enhanced HER performance of nickel sulphide electrodes. However, beyond these structural and electronic modifications, it is also essential to assess whether electrochemical activation influences the fundamental HER mechanism. To investigate this, the Tafel slopes before and after activation were analyzed (Figures 3b and 5), providing insight into possible modifications in the rate‐determining step within the Volmer–Heyrovsky–Tafel mechanism [79]. These modifications are visualized in Figure 5, wherein the boxed regions in the plots highlight the overpotential range used to extract the Tafel values.

The substantial reduction in Tafel slopes across all samples indicates a significant improvement in HER kinetics upon electrochemical activation. Before activation, the high Tafel slopes (122–187 mV·dec^−1^) suggest that the Volmer step is the rate‐determining step (RDS) in the HER mechanism, indicating sluggish kinetics associated with hydrogen adsorption on the electrode surface. After activation, the Tafel slopes decrease substantially (63–81 mV·dec^−1^), suggesting a transition in the RDS, where the Heyrovsky step becomes more dominant [80, 81]. This shift in catalytic performance is attributed to structural and compositional modifications induced by electrochemical activation, particularly Sulfur leaching and the exposure of undercoordinated Ni sites, which lead to the formation of Ni‐rich surfaces. Several studies have highlighted the beneficial role of Sulfur vacancies in optimizing the electronic structure of nickel Sulfides. D. Jia et al. [35] demonstrated that the presence of Sulfur vacancies in nickel Sulfides results in an upward shift of the d‐band center of Ni atoms toward the Fermi level, leading to enhanced electronic conductivity and catalytic activity. Similarly, Hu et al. [82] confirmed that a positive shift in the d‐band center occurs upon Sulfur removal, further improving charge‐transfer kinetics. A higher d‐band center energy strengthens Ni–H interactions, promoting stronger hydrogen adsorption at the Ni surface, which is a critical step in the Volmer mechanism. Following electrochemical activation, this effect ensures more efficient hydrogen adsorption at the exposed Ni sites, facilitating the initial H_2_O dissociation and hydrogen binding. In addition, the phase transformation to the semi‐metallic Ni_3_S_2_ phase, which exhibits higher electrical conductivity, is key in lowering kinetic barriers and facilitating more efficient electron transfer during HER [24, 83]. These modifications collectively accelerate HER kinetics, explaining the improvements in Tafel slopes and charge transfer resistance post‐activation.

### 3.3 Impact of Sulfur Leaching on Long‐Term Stability

**FIGURE 6:**
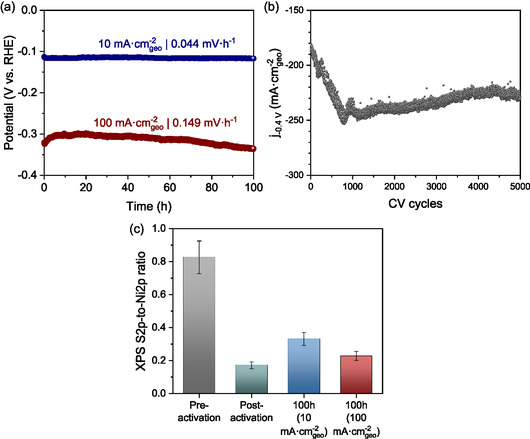
(a) Constant‐current polarization curves of the activated Ni_3_S_2_ sample at 10 and 100  mA·cm_geo_^−2^ over 100 h in 1.0 M KOH. (b) Activation profile of Ni_3_S_2_ subjected to 5000 CV sweeps in the potential range of −0.4 to 0.0 V vs. RHE at a scan rate of 50 mV·s^−1^ in 1.0 M KOH. (c) XPS‐derived S2p‐to‐Ni2p ratio of Ni_3_S_2_ before activation, after 500 CV sweeps, and after 100 h of constant‐current stability testing at 10 and 100 mA·cm_geo_^−2^. Error bars in Figure 6c reflect combined uncertainties from measurements at three distinct locations on each sample and the error margins associated with peak area fitting in the XPS analysis.

The role of Sulfur leaching and phase transformation in enhancing HER activity and modifying electrochemical properties has been discussed. However, a key concern is whether these structural changes have a detrimental effect on the long‐term stability of the catalyst. Sulfur loss, in particular, has been associated with the potential degradation of catalytic performance over extended operation [84]. To evaluate the stability of the most active catalyst, Ni_3_S_2_, which demonstrated the best electrochemical properties, was subjected to constant‐current polarization tests following electrochemical activation for 100 h. To ensure a realistic assessment, two commonly used current densities from literature, 10 and 100 mA·cm_geo_^−2^, were selected (Figure 6a). As shown, Ni_3_S_2_ exhibits excellent long‐term stability, with a minimal degradation rate of 0.044 mV·h^−1^ at 10 mA·cm_geo_^−2^ and 0.149 mV·h^−1^ at 100 mA·cm_geo_^−2^. These results suggest that despite Sulfur leaching and structural modifications, the catalyst remains stable under prolonged electrochemical conditions.

To further investigate the extent and durability of the electrochemical activation process, the Ni_3_S_2_ electrode was subjected to an extended CV cycling test for 5000 CV cycles in the same potential range of −0.4 to 0.0 V versus RHE, significantly exceeding the typical 500‐cycle activation protocol (Figure 6b). This experiment aimed to assess how long the activation persists and whether the catalyst remains stable over prolonged cycling. Initially, the sample exhibited a current density of −180 mA·cm_geo_^−2^ at −0.4 V versus RHE, which steadily increased during the initial activation phase. After ≈800 CV cycles, the current density reached its maximum value of −255 mA·cm_geo_^−2^, indicating a progressive improvement in HER activity. Beyond this point, the current density started to stabilize within the range of −250 to −225 mA·cm_geo_^−2^ for the remainder of the CV cycles, suggesting that the electrode undergoes an activation‐saturation process followed by a steady‐state operation. These results confirm that the electrochemical activation process is not only effective in enhancing HER performance but also reaches a self‐limiting state, beyond which further activation does not significantly improve activity. More importantly, after reaching its optimal activity, Ni_3_S_2_ maintains a relatively stable current response, reinforcing its durability under extended electrochemical cycling.

To further investigate the structural stability of Ni_3_S_2_ after extended HER operation, XPS analysis was conducted following the 100‐hour stability tests at 10 and 100 mA·cm_geo_^−2^, and the S 2p‐to‐Ni 2p ratio was compared to previous activation results (Figure 6c). As previously determined, the initial activation process (500 CV cycles) resulted in significant Sulfur leaching, reducing the S 2p‐to‐Ni 2p ratio from 0.83 to 0.17. Following 100 h of constant‐current polarization, the S 2p‐to‐Ni 2p ratio increased slightly to 0.33 and 0.23 for the 10 and 100 mA·cm_geo_^−2^ stability tests, respectively. This observation suggests that while Sulfur loss initially occurs during activation, it does not continuously degrade over prolonged operation. Instead, a stabilization effect is observed, likely due to the reorganization of residual Sulfur species or possible re‐adsorption of Sulfur‐containing species from the electrolyte. Notably, the slightly lower ratio at 100 mA·cm_geo_^−2^ compared to 10 mA·cm_geo_^−2^ indicates that higher current densities induce slightly more Sulfur depletion, possibly due to stronger electrochemical driving forces. However, the difference remains relatively small, demonstrating that even at higher HER operating conditions, Ni_3_S_2_ retains its structural stability. The most significant finding is that the S 2p‐to‐Ni 2p ratio did not decrease further below 0.17 after 100 h of operation, confirming that the catalyst does not undergo progressive Sulfur depletion over time. Instead, it reaches a stable Sulfur composition, which is critical for preserving its electrochemical activity and durability. These combined results, illustrated in Figure 6a–c, confirm that the initial Sulfur loss plays a key role in activation but does not compromise the catalyst's long‐term durability, directly supporting the conclusion that Ni_3_S_2_ is both high‐performing and structurally robust under prolonged HER conditions.

## 4 Conclusions

This study provides a comprehensive investigation into the electrochemical activation, structural evolution, and long‐term stability of different nickel sulphide electrocatalysts (NiS, NiS_2_, Ni_3_S_2_, and Ni_3_S_4_) for the hydrogen evolution reaction in alkaline media. Prolonged electrochemical activation through CV cycling results in a phase transformation in all nickel sulphide phases toward Ni_3_S_2_, which is identified as the most HER‐active phase. This phase transformation is accompanied by selective Sulfur leaching, with the XPS S 2p‐to‐Ni 2p ratio decreasing from 0.82 to 1.45 to 0.17–0.27, and by structural modifications that reduce charge–transfer resistance (1.4–2.4‐fold), increase electrochemical surface area (1.2–3.0‐fold), and enhance HER performance by up to ∼2‐fold in certain cases. In addition, this structural modification post‐activation induces a reduction in Tafel slopes from 122 to 187 to 63–81 mV·dec^−1^, suggesting a transition in the HER mechanism, shifting from a Volmer‐limited pathway to a Heyrovsky‐dominant process, reflecting the improved hydrogen adsorption–desorption equilibrium following phase transformation and electrochemical restructuring. Despite initial Sulfur leaching, the long‐term stability of Ni_3_S_2_ was well preserved, as confirmed through constant‐current polarization tests with a low degradation rate of 0.149 mV·h^−1^ at 100 mA·cm_geo_^−2^. While Sulfur loss is often considered a potential degradation factor, our findings indicate that Sulfur depletion is self‐limiting, reaching a stable Sulfur‐to‐nickel ratio of 0.25 ± 0.8 over prolonged HER operation. The absence of continuous Sulfur loss ensures that Ni_3_S_2_ remains electrochemically active and structurally stable, making it efficient for prolonged HER applications. The findings provide insights into the activation mechanisms of nickel Sulfides, highlighting that Sulfur leaching and structural reorganization can be leveraged to enhance catalytic performance without compromising stability.

## Supporting Information

Additional supporting information can be found online in the Supporting Information section. **Supporting** **Fig.** **S1:** Grazing‐incidence X‐ray diffraction (GIXRD) patterns of the as‐synthesized nickel sulphide phases (top) and corresponding reference patterns from the PDF database (bottom). **Supporting Fig.** **S2:** XPS survey spectra of NiS before and after electrochemical activation. **Supporting Fig.** **S3:** XPS survey spectra of NiS_2_ before and after electrochemical activation. **Supporting Fig.** **S4:** XPS survey spectra of Ni_3_S_2_ before and after electrochemical activation. **Supporting Fig.** **S5:** XPS survey spectra of Ni_3_S_4_ before and after electrochemical activation. **Supporting Fig.** **S6:** High‐resolution XPS O 1s spectra of (a) NiS, (b) NiS_2_, (c) Ni_3_S_2_, and (d) Ni_3_S_4_ before and after electrochemical activation. **Supporting Fig.** **S7:** Low magnification SEM images of (a,b) Ni_3_S_2_, (c,d) NiS (e,f) Ni_3_S_4_, and (g,h) NiS_2_ electrodes. Panels (a,c,e,g) correspond to the samples before HER, while panels (b,d,f,h) show the same samples after HER. **Supporting Fig.** **S8:** High‐magnification SEM images of (a,b) Ni_3_S_2_, (c,d) NiS (e,f) Ni_3_S_4_, and (g,h) NiS_2_ electrodes. Panels (a,c,e,g) correspond to the samples before HER, while panels (b,d,f,h) show the same samples after HER. **Supporting Fig.** **S9:** Repeated CV plots of (a) NiS, (b) NiS_2_, (c) Ni_3_S_2_, and (d) Ni_3_S_4_ samples before electrochemical activation recorded at various scan rates within a potential window of 0.1–0.2 V vs. RHE, excluding Faradaic processes. (e) The average variance between anodic and cathodic currents at 0.15 V vs. RHE as a function of scan rate. (f) ECSA of various nickel sulphide samples before electrochemical activation. **Supporting Fig.** **S10:** Repeated CV plots of (a) NiS, (b) NiS_2_, (c) Ni_3_S_2_, and (d) Ni_3_S_4_ samples after electrochemical activation recorded at various scan rates within a potential window of 0.1–0.2 V vs. RHE, excluding Faradaic processes. (e) The average variance between anodic and cathodic currents at 0.15 V vs. RHE as a function of scan rate. (f) ECSA of various nickel sulphide samples after electrochemical activation. **Supporting Fig.** **S11:** EIS Nyquist plots of (a) NiS, (b) NiS_2_, (c) Ni_3_S_2_, and (d) Ni_3_S_4_ samples before and after electrochemical activation. **Supporting Table S1**: Summary of Raman‐active vibrational modes and corresponding peak positions of nickel sulphide phases.

## Conflicts of Interest

The authors declare no conflicts of interest.

## Supporting information

Supplementary Material

## Data Availability

The dataset supporting this study is available in Zenodo at https://doi.org/10.5281/zenodo.16920699 [85].
