# Supplementary material for: Structural and Electrochemical Evolution of Nickel Sulfides During Alkaline Hydrogen Evolution Reaction
Source: ChemSusChem. 2025 Dec 19;19(7):e202501880. doi: 10.1002/cssc.202501880 (PMC13021358; doi:10.1002/cssc.202501880)
Supplement: Supplementary file 1 — Supplementary Material [file CSSC-19-e202501880-s001.pdf]

Structural and Electrochemical Evolution of Nickel Sulphides during Alkaline Hydrogen Evolution Reaction

Sina Haghverdi Khamene[1,2], Noëlle van Dalen[1], Mariadriana Creatore[1,3], Mihalis N. Tsampas[2]

1 Department of Applied Physics and Science Education, Eindhoven University of Technology, 5600 MB Eindhoven, the Netherlands

2 DIFFER – Dutch Institute For Fundamental Energy Research, 5612 AJ Eindhoven, the Netherlands

3 Eindhoven Institute for Renewable Energy Systems (EIRES), 5600 MB Eindhoven, the Netherlands

Table S1. Summary of Raman-active vibrational modes and corresponding peak positions of nickel sulphide phases.

Phase	Raman shift (cm^{-1})	Vibrational modes	Reference(s)
**Ni_3S_2**	188, 201, 224, 304, 325, 350	2×A1, 4×E	[1,2]
**β-NiS**	146, 221, 244, 284, 298, 349, 371	various	[3,4]
**Ni_3S_4**	224, 287, 337, 380	Eg, 2×Tg, Ag	[5,6]
**NiS_2**	275, 285, 480	Tg, Eg, Ag	[3,7]

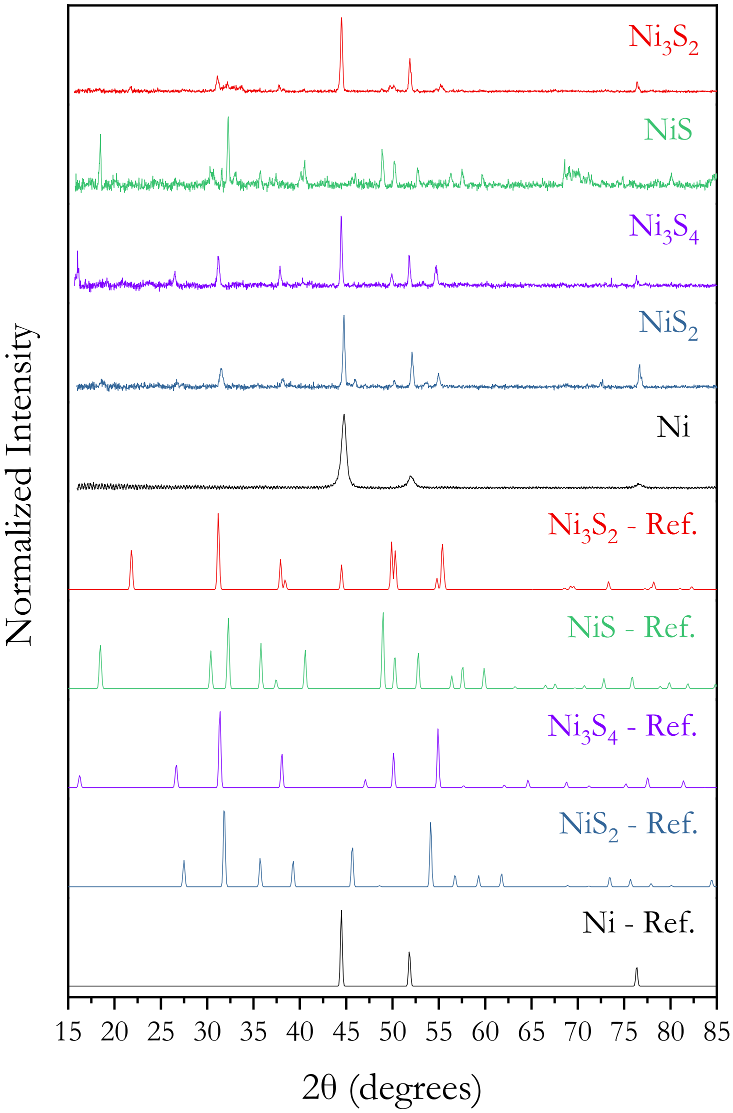

Figure S1. Grazing-incidence X-ray diffraction (GIXRD) patterns of the as-synthesized nickel sulphide phases (top) and corresponding reference patterns from the PDF database (bottom).

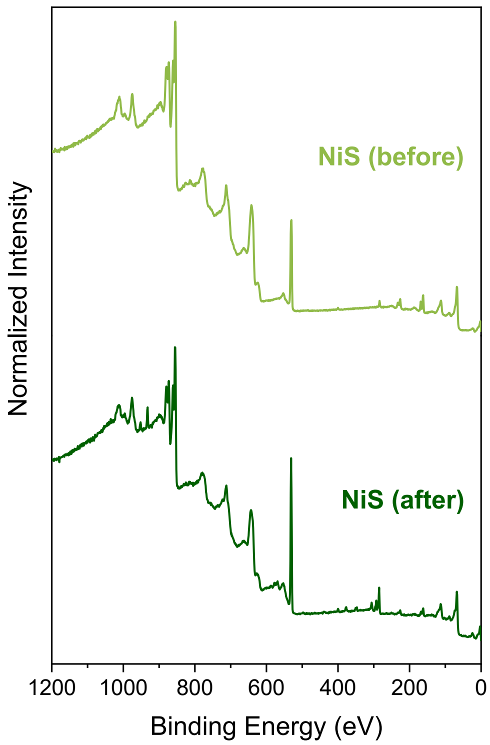

Figure S2. XPS survey spectra of NiS before and after electrochemical activation.

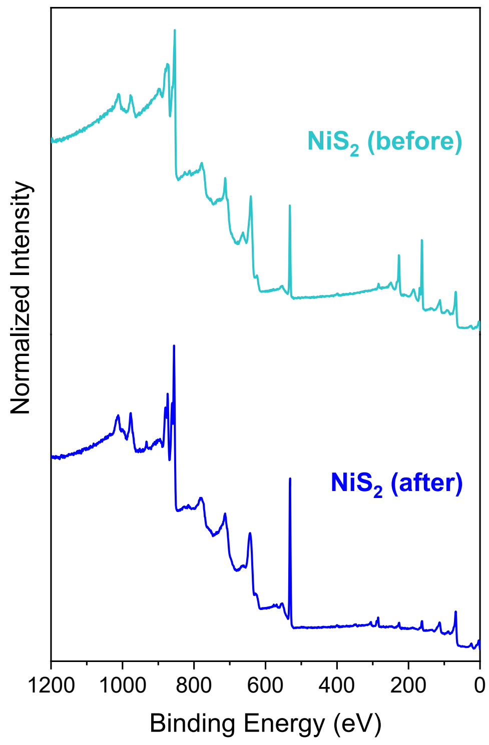

Figure S3. XPS survey spectra of NiS_2 before and after electrochemical activation.

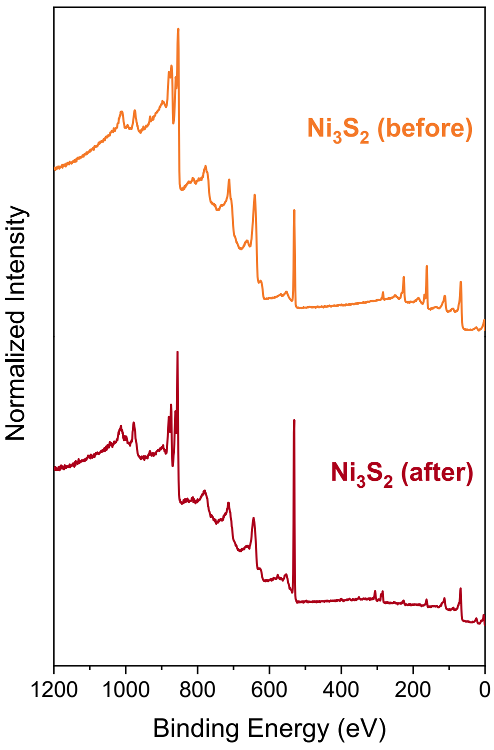

Figure S4. XPS survey spectra of Ni_3S_2 before and after electrochemical activation.

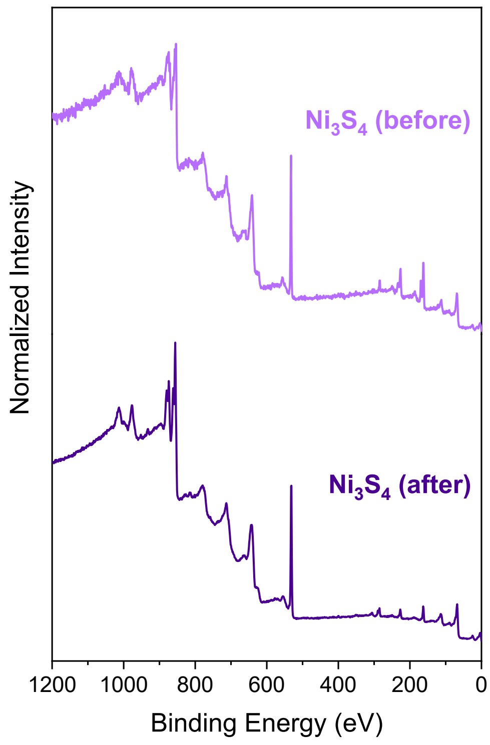

Figure S5. XPS survey spectra of Ni_3S_4 before and after electrochemical activation.

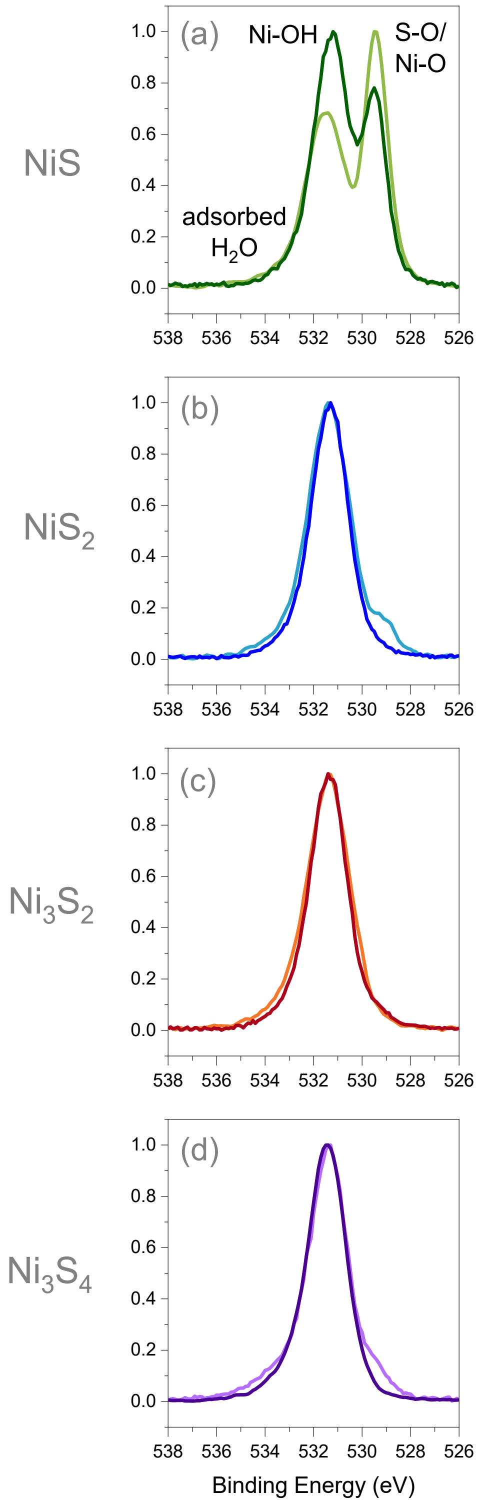

Figure S6. High-resolution XPS O 1s spectra of (a) NiS, (b) NiS_2, (c) Ni_3S_2, and (d) Ni_3S_4 before and after electrochemical activation.

Analysis of Surface Oxygen Species via O 1s XPS Before and After HER

The high-resolution O 1s XPS spectra (Figure S5) provide insights into the surface oxygen species present in the samples before and after HER. Across all samples, the spectra show a primary peak centered at ~529.5 eV, corresponding to metal-oxygen (Ni-O) bonds, which likely arise from the formation of NiO or nickel oxysulphides due to partial surface oxidation upon exposure to air. In addition, a secondary peak at ~531.2 eV, along with peak broadening at higher binding energies, is attributed to $Ni(OH)_2$ and surface-adsorbed hydroxyl species, indicating the presence of surface hydration or hydroxylation. The higher intensity of the Ni-O/S-O peak in the NiS sample can be attributed to differences in synthesis temperature, as higher synthesis temperatures increase the likelihood of oxidation. After HER, the spectra reveal a shift from binding energies characteristic of S–O and Ni-O bonds to those associated with Ni-OH species, confirming Ni 2p results. In most samples, this transformation appears complete, as evidenced by the predominance of Ni-OH signals in the O 1s spectra. However, the NiS sample displays only a partial conversion, suggesting that some S–O and Ni–O species remain untransformed. This is supported by the fact that $Ni(OH)_2$ formation is a common occurrence during electrochemical processes in alkaline media due to direct exposure to the electrolyte.

Discussion on Sulphur Leaching Pathways Under Cathodic Conditions

In addition, XPS analysis revealed a consistent decrease in the S/Ni ratio across all samples after HER cycling, indicating sulphur leaching from the nickel sulphide phases. As the experiments were conducted in 1.0 M KOH continuously purged with N_2 and under strictly cathodic conditions, the oxidation of sulphide (S^{2-}) to sulphate (SO_4^{2-}) is thermodynamically unfavorable due to the absence of oxidizing agents such as O_2 or anodic potentials. While trace amounts of residual oxygen may remain despite purging, their influence on sulphur oxidation is expected to be negligible. The leached sulphur is therefore most likely present in the electrolyte as dissolved sulphide species (S^{2-}/HS^{-}).

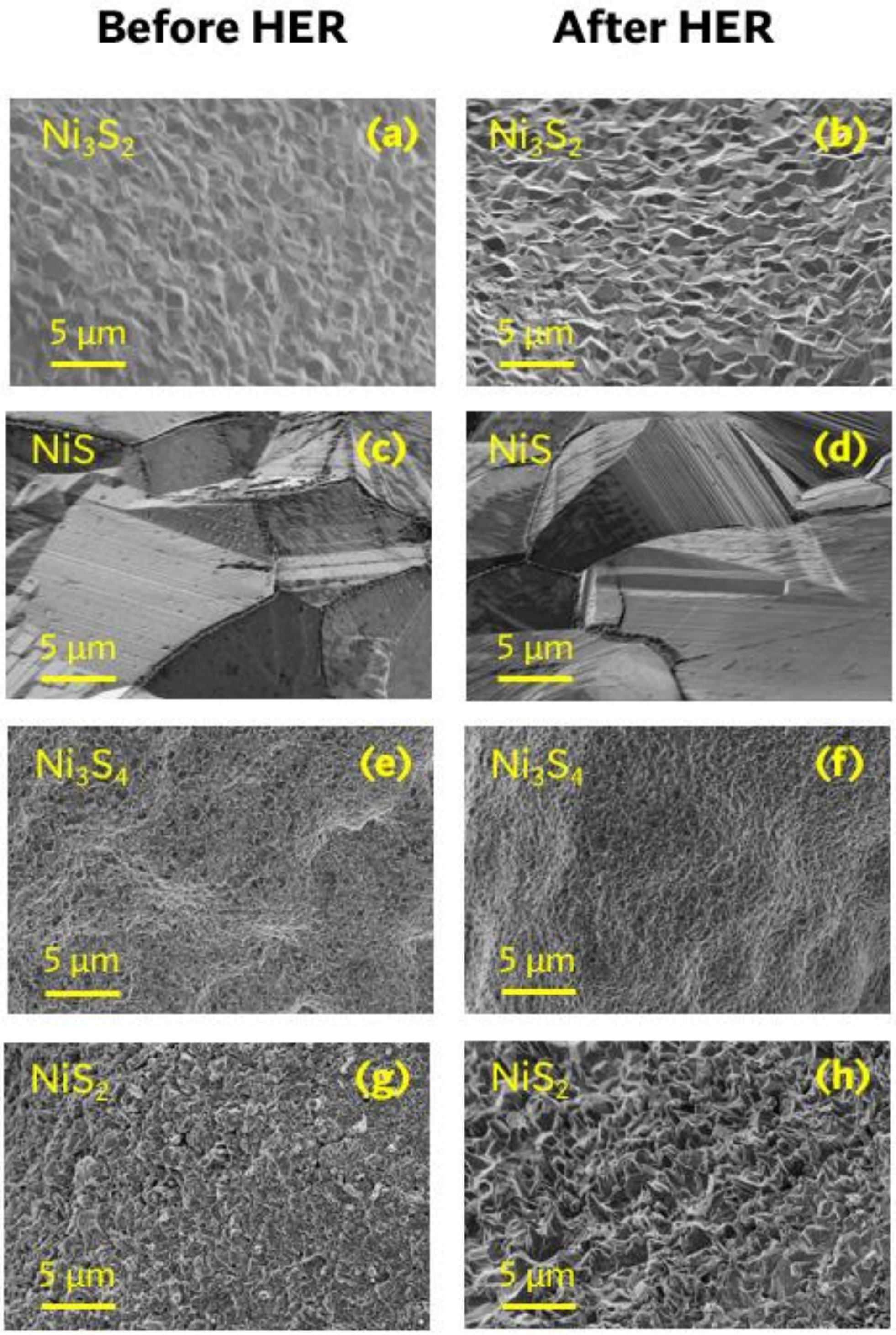

Figure S7. Low magnification SEM images of (a–b) Ni_3S_2, (c–d) NiS (e–f) Ni_3S_4, and (g–h) NiS_2 electrodes. Panels (a, c, e, g) correspond to the samples before HER, while panels (b, d, f, h) show the same samples after HER.

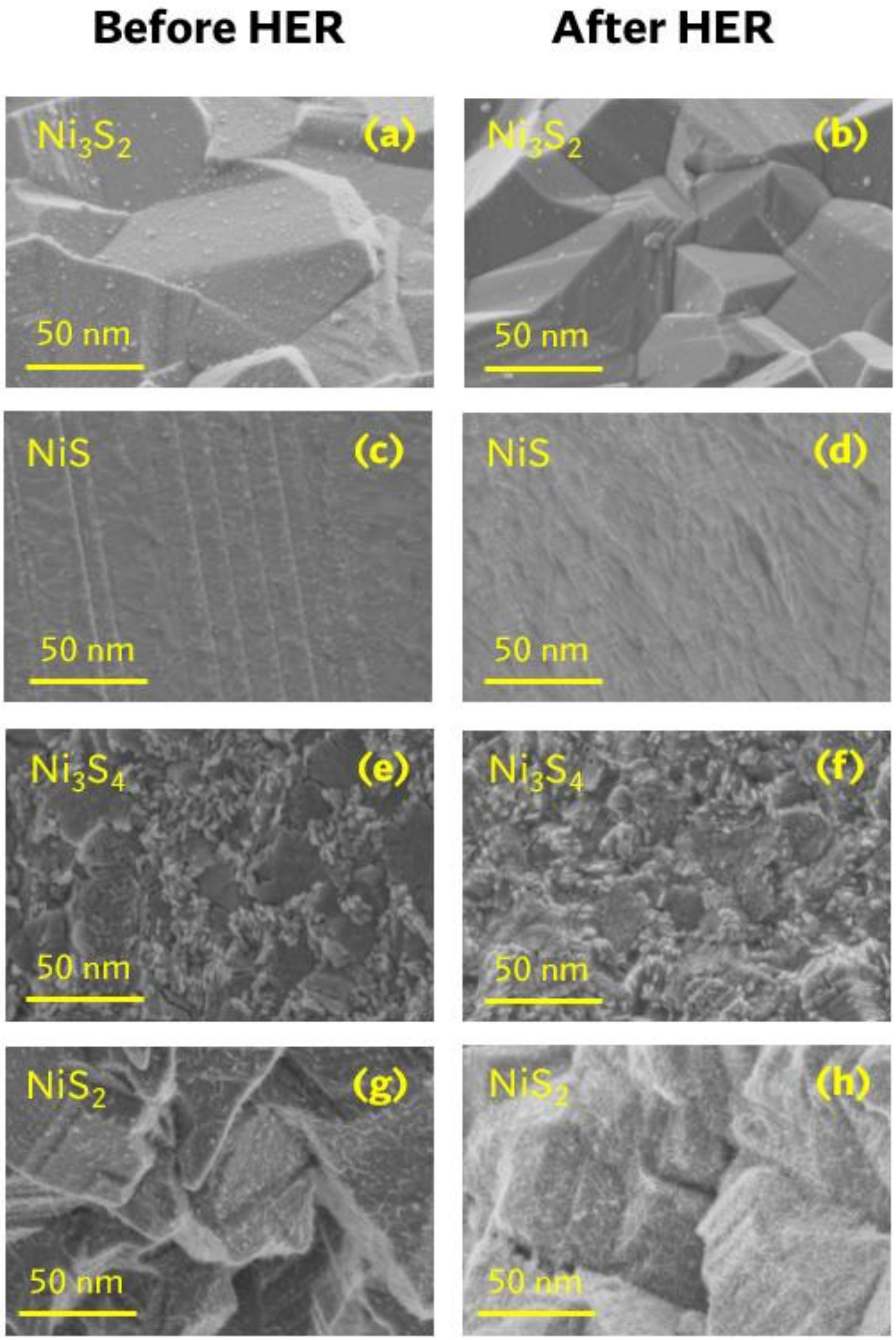

Figure S8. High magnification SEM images of (a–b) Ni_3S_2, (c–d) NiS (e–f) Ni_3S_4, and (g–h) NiS_2 electrodes. Panels (a, c, e, g) correspond to the samples before HER, while panels (b, d, f, h) show the same samples after HER.

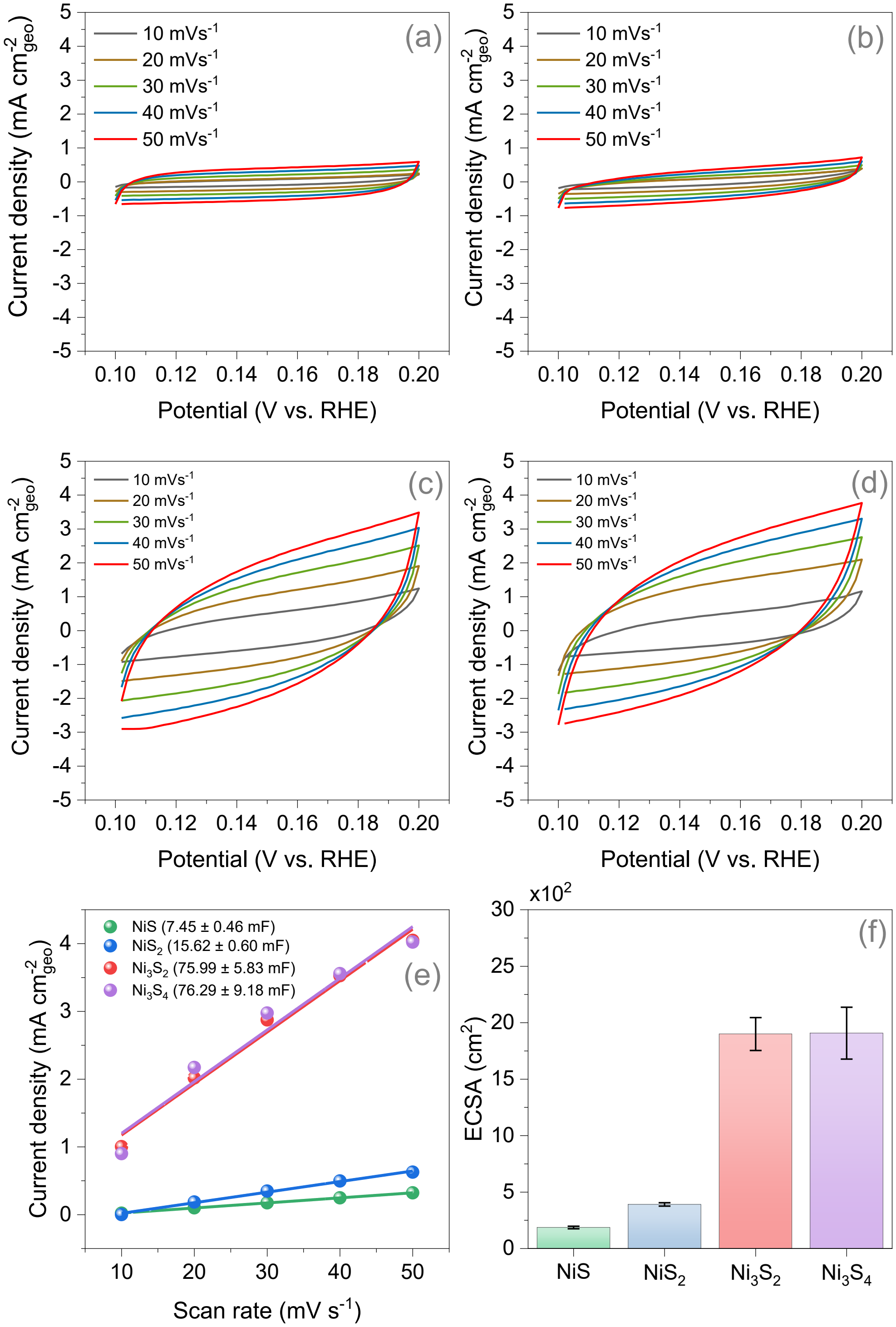

Figure S9. Repeated CV plots of (a) NiS, (b) NiS_2, (c) Ni_3S_2, and (d) Ni_3S_4 samples before electrochemical activation recorded at various scan rates within a potential window of 0.1 to 0.2 V vs. RHE, excluding Faradaic processes. (e) The average variance between anodic and cathodic currents at 0.15 V vs. RHE as a function of scan rate. (f) ECSA of various nickel sulphide samples before electrochemical activation.

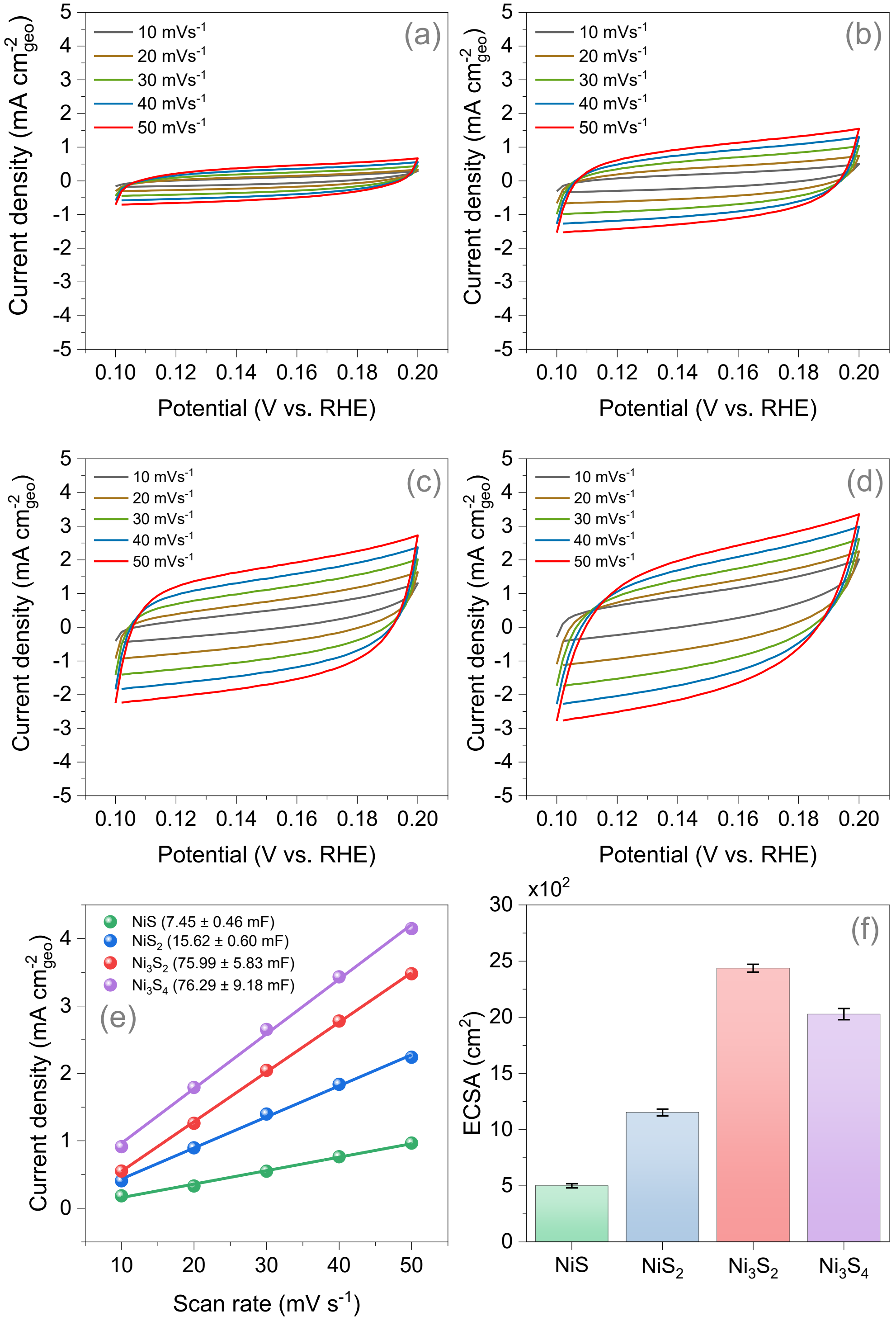

Figure S10. Repeated CV plots of (a) NiS, (b) NiS_2, (c) Ni_3S_2, and (d) Ni_3S_4 samples after electrochemical activation recorded at various scan rates within a potential window of 0.1 to 0.2 V vs. RHE, excluding Faradaic processes. (e) The average variance between anodic and cathodic currents at 0.15 V vs. RHE as a function of scan rate. (f) ECSA of various nickel sulphide samples after electrochemical activation.

The ECSA was estimated using ECSA = C_{dl} / C*. Reported values of the specific capacitance (C*) for Ni-based sulphides and oxides in alkaline media vary considerably due to differences in composition, surface roughness, and electrolyte conditioning. Owing to the limited and inconsistent data available in the literature, a representative constant value of 40 $\mu F \cdot cm^{-2}$, typical for metal sulphides in alkaline media, was employed to calculate the absolute ECSA values. However, to minimise the influence of potential inaccuracies in this assumption, the analysis in this work primarily relies on the ECSA evolution ratio before and after HER activation, for which the impact of variations in specific capacitance is negligible.

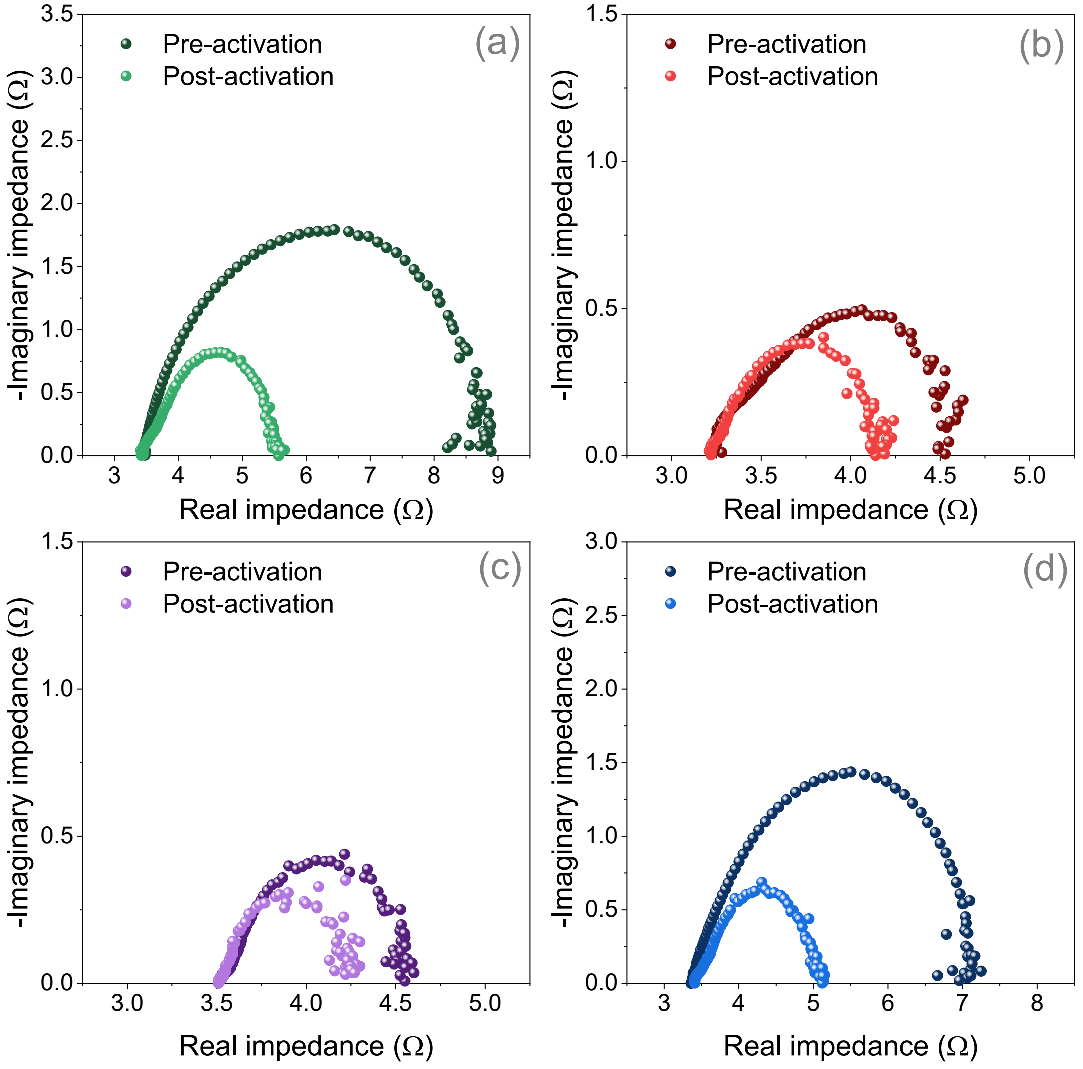

Figure S11. EIS Nyquist plots of (a) NiS, (b) NiS_2, (c) Ni_3S_2, and (d) Ni_3S_4 samples before and after electrochemical activation.

Theoretical Basis for Ni_3S_2's Superior HER Performance

Ni_3S_2 is considered the most electrocatalytically active phase among nickel sulphides in alkaline hydrogen evolution, thanks to its unique electronic structure and optimal adsorption energetics. DFT studies reveal that Ni_3S_2 is intrinsically metallic, in which its Fermi level crosses Ni 3d bands, whereas NiS_2 and NiS are semiconductors with band gaps of ~0.4 eV and ~1.1 eV, respectively.[8] This near-metallic conductivity of Ni_3S_2 facilitates electron transfer during HER, resulting in lower charge-transfer resistance and faster reaction kinetics. Moreover, the Ni-S framework in Ni_3S_2 features a higher Ni-to-S ratio, meaning more Ni active sites are available on the surface. Consequently, Ni_3S_2 offers a higher density of HER active sites compared to the rest of the Ni_xS_y phases.[8] Beyond conductivity, Ni_3S_2 provides an ideal hydrogen binding strength. *Ab initio* calculations show that Ni_3S_2 has a near-thermoneutral Gibbs free energy for hydrogen adsorption, which is much closer to the Sabatier optimum than for NiS_2 or NiS.[9] Accordingly, the Ni_3S_2 catalyst is able to bind H atoms neither too weakly nor too strongly, facilitating efficient H-H bond formation and release of H_2. This synergistic effect explains why Ni_3S_2 exhibits intrinsically higher catalytic activity for alkaline HER than other nickel sulphide phases.

Role of Electrolyte Purity in Long-Term Catalyst Durability

Electrolyte purification plays a critical role in determining catalyst durability in alkaline HER systems. Carbonate species (CO_3^{2-}), formed from CO_2 absorption into KOH, can reduce ionic conductivity, poison active sites, and precipitate within porous structures, all of which accelerate catalyst degradation. While our experiments were conducted in CO_2-free conditions using continuously N_2-purged 1.0 M KOH, we note that in practical systems, the presence of carbonate impurities can hinder long-term performance. Prior studies[10–12] have shown that nickel-based catalysts in purified KOH maintain structural integrity and stable activity far longer than those exposed to carbonate-rich or contaminated environments. Therefore, although we did not include CO_3^{2-} in our experimental design, its influence on practical catalyst stability is well established.

References

(1) Cheng, Z.; Abernathy, H.; Liu, M. Raman Spectroscopy of Nickel Sulfide Ni_3S_2. *Journal of Physical Chemistry C* 2007, *111* (49), 17997–18000. https://doi.org/10.1021/JP0770209.

(2) Wang, Z.; Shen, S.; Lin, Z.; Tao, W.; Zhang, Q.; Meng, F.; Gu, L.; Zhong, W.; Wang, Z.; Shen, S.; Lin, Z.; Tao, W.; Zhong, W.; Zhang, Q.; Meng, F.; Gu, L. Regulating the Local Spin State and Band Structure in Ni_3S_2 Nanosheet for Improved Oxygen Evolution Activity. *Adv Funct Mater* 2022, *32* (18), 2112832. https://doi.org/10.1002/ADFM.202112832.

(3) Sun, Y.; Wu, J.; Zhang, Z.; Liao, Q.; Zhang, S.; Wang, X.; Xie, Y.; Ma, K.; Kang, Z.; Zhang, Y. Phase Reconfiguration of Multivalent Nickel Sulfides in Hydrogen Evolution. *Energy Environ Sci* 2022, *15* (2), 633–644. https://doi.org/10.1039/D1EE02985A.

(4) Bishop, D. W.; Thomas, P. S.; Ray, A. S. Raman Spectra of Nickel(II) Sulfide. *Mater Res Bull* 1998, *33* (9), 1303–1306. https://doi.org/10.1016/S0025-5408(98)00121-4.

(5) Yin, P. F.; Sun, L. L.; Zhou, C.; Sun, Y. H.; Han, X. Y.; Deng, C. R. Synthesis, Characterization and Magnetic Property of 3D Flower-like Nickel Sulphide Nanocrystals through Decomposing Bis(Thiourea) Nickel(II) Chloride Crystals. *Bulletin of Materials Science* 2015, *38* (1), 95–99. https://doi.org/10.1007/S12034-014-0815-6.

(6) Cheng, Z.; Liu, M. Characterization of Sulfur Poisoning of Ni–YSZ Anodes for Solid Oxide Fuel Cells Using in Situ Raman Microspectroscopy. *Solid State Ion* 2007, *178* (13–14), 925–935. https://doi.org/10.1016/J.SSI.2007.04.004.

(7) Dai, C.; Li, B.; Li, J.; Zhao, B.; Wu, R.; Ma, H.; Duan, X. Controllable Synthesis of NiS and NiS_2 Nanoplates by Chemical Vapor Deposition. *Nano Res* 2020, *13* (9), 2506–2511. https://doi.org/10.1007/S12274-020-2887-5.

(8) Jiang, N.; Tang, Q.; Sheng, M.; You, B.; Jiang, D. E.; Sun, Y. Nickel Sulfides for Electrocatalytic Hydrogen Evolution under Alkaline Conditions: A Case Study of Crystalline NiS, NiS_2, and Ni_3S_2 Nanoparticles. *Catal Sci Technol* 2016, *6* (4), 1077–1084. https://doi.org/10.1039/C5CY01111F.

(9) Zheng, X.; Han, X.; Zhang, Y.; Wang, J.; Zhong, C.; Deng, Y.; Hu, W. Controllable Synthesis of Nickel Sulfide Nanocatalysts and Their Phase-Dependent Performance for Overall Water Splitting. *Nanoscale* 2019, *11* (12), 5646–5654. https://doi.org/10.1039/C8NR09902B.

(10) Becker, H.; Murawski, J.; Shinde, D. V.; Stephens, I. E. L.; Hinds, G.; Smith, G. Impact of Impurities on Water Electrolysis: A Review. *Sustain Energy Fuels* 2023, *7* (7), 1565–1603. https://doi.org/10.1039/D2SE01517J.

(11) Spanos, I.; Tesch, M. F.; Yu, M.; Tüysüz, H.; Zhang, J.; Feng, X.; Müllen, K.; Schlögl, R.; Mechler, A. K. Facile Protocol for Alkaline Electrolyte Purification and Its Influence on a Ni–Co Oxide Catalyst for the Oxygen Evolution Reaction. *ACS Catal* 2019, *9* (9), 8165–8170. https://doi.org/10.1021/ACSCATAL.9B01940.

(12) d'Amore-Domenech, R.; Carrillo, I.; Navarro, E.; Leo, T. J. Alkaline Electrolysis for Hydrogen Production at Sea: Perspectives on Economic Performance. *Energies 2023, Vol. 16, Page 4033* 2023, *16* (10), 4033. https://doi.org/10.3390/EN16104033.
